# Grazing versus housing in native dairy goats: Impacts on milk yield, composition, and conjugated linoleic acid enrichment under arid conditions

**DOI:** 10.14202/vetworld.2025.4093-4104

**Published:** 2025-12-27

**Authors:** María Teresa Trejo-López, Omar Hernández-Mendo, Lorenzo Danilo Granados-Rivera, Glafiro Torres-Hernández, Jorge Alonso Maldonado-Jáquez, David Hernández-Sánchez

**Affiliations:** 1 Programa de Ganadería, Colegio de Postgraduados Campus Montecillo, Texcoco, Texcoco de Mora, 56230, Mexico; 2 Department of Animal Nutrition, National Institute of Forestry, Agricultural, and Livestock Research. General Terán Experimental Field. General Terán, Nuevo León, 67400, Mexico; 3 Department of Animal Genetics, National Institute of Forestry, Agricultural and Livestock Research. La Laguna Experimental Field. Matamoros, Coahuila, 27440, Mexico

**Keywords:** conjugated linoleic acid, fatty acids, grazing, housing system, milk quality, native goats

## Abstract

**Background and Aim:**

Milk yield and composition in goats are heavily influenced by feeding and management practices, especially in arid areas where forage availability varies. Native goats in northern Mexico are well adapted to tough environments, yet there is limited evidence on how different production systems impact their milk quality. This study aimed to compare milk yield, chemical makeup, and the fatty acid (FA) profile, particularly conjugated linoleic acid (CLA), of early-lactation native goats managed under grazing and housed systems.

**Materials and Methods:**

Ten clinically healthy native goats in early-lactation were randomly assigned to two treatments: grazing (n = 5) and housed (n = 5). Housed goats received a mixed ration, while grazing goats foraged daily on native rangeland. Milk yield was recorded weekly, and 100 mL composite milk samples were analyzed for fat, protein, lactose, and FA profile using infrared spectrophotometry and gas chromatography. The experimental period included 14 days of adaptation and 42 days of data collection. Data were analyzed using a completely randomized design with repeated-measures in PROC MIXED (SAS v9.4), and Tukey’s test was applied for comparisons (p < 0.05).

**Results:**

Housed goats showed significantly higher (p < 0.05) daily milk yield (1.41 vs. 0.81 kg per day) and fat-corrected milk (1.22 vs. 0.83 kg per day). Protein and lactose concentrations were also greater in housed goats. In contrast, grazing goats produced milk with higher fat concentration (+42.3%; p = 0.0238) and a more favorable FA profile, including increased unsaturated FAs such as oleic acid (C18:1 c9), α-linolenic acid (C18:3 n-3), and cis-9, trans-11 CLA (p = 0.0009). Grazing also decreased medium-chain saturated FAs while increasing long-chain FAs and total monounsaturated FAs.

**Conclusion:**

Grazing boosts the nutritional quality of milk from native goats by increasing unsaturated FAs and significantly raising CLA levels, though it comes with a reduction in milk volume. These results underscore the importance of native goats and extensive grazing systems for producing nutrient-rich milk in arid areas, supporting both sustainability and potential markets for functional foods.

## INTRODUCTION

Goat dairying supports millions of smallholders in arid and semi-arid areas where native genotypes perform better than exotic breeds under heat stress and feed shortages. In Mexico’s Comarca Lagunera, the country’s primary goat-milk basin, local herds produced 58.9 million liters in 2023, accounting for one-third of the national output [1, 2]. About 70% of these goats graze native rangeland during the day and are penned at night, while the remaining 30% are kept in continuous confinement and fed concentrate-based diets [3]. As a result, the production system influences nutrient supply, which ultimately affects milk yield and composition.

The dietary forage-to-concentrate ratio significantly influences fatty acid (FA) kinetics in milk. Pasture high in α-linolenic acid boosts milk cis-9, trans-11 conjugated linoleic acid (CLA) and n-3 polyunsaturated FAs, while concentrate feeding increases total milk production but leads to a more saturated FA profile [4, 5]. However, most existing evidence comes from high-yielding imported breeds raised indoors, and data on native goats in desert environments with contrasting management systems are limited. Only two Mexican studies have examined the milk FA profile of local goats, both focusing on late-lactation and mixed-feeding conditions [6, 7]. No research has simultaneously evaluated early-lactation milk yield, proximate composition, and detailed FA spectra in native does under strictly defined grazing versus housing systems.

The health significance of this data is substantial. A meta-analysis of human trials showed that a daily intake of 3 g cis-9, trans-11 CLA lowered fasting blood sugar by 4% and decreased tumor-proliferation markers by 7% [8]. Goat milk can provide up to 60 mg of CLA per 100 mL when grazing is the main feed source, almost double the level found in stall-fed goats [4].

Despite the significance of native goats in arid and semi-arid regions, there is limited knowledge of how different production systems affect their milk traits. Most existing research on milk yield, chemical makeup, and FA profiles has come from high-producing exotic breeds kept under intensive indoor management, leaving a major gap in understanding for locally adapted animals. In Mexico, where about 70% of native goats rely on rangeland grazing, studies on their milk FA composition are scarce and mostly focused on late-lactation or mixed-feeding methods, which hampers the interpretation of system effects. No previous studies have simultaneously examined early-lactation milk yield, basic composition, and detailed FA spectra (including cis-9, trans-11 CLA) in native goats subjected to specific grazing and housing systems under desert conditions. This lack of integrated, system-level data limits the ability to create nutritional strategies, functional dairy products, and sustainability guidelines tailored to native goat production in arid regions.

This study aimed to generate comprehensive, system-specific evidence on how grazing and housing management affect milk production traits in native goats during early-lactation. Specifically, the objectives were to: (i) compare daily milk yield and fat-corrected milk (FCM) output between grazing and housed goats; (ii) assess differences in the chemical composition of milk, including fat, protein, and lactose; and (iii) characterize the complete milk FA profile, with emphasis on health-promoting components such as cis-9, trans-11 CLA and n-3 polyunsaturated FAs. By combining production metrics with detailed FA spectra, the study aimed to clarify the trade-offs between milk volume and nutritional quality across systems. The resulting evidence is intended to support decision-making for producers, promote value-added dairy products, and strengthen sustainable goat production in arid regions.

## MATERIALS AND METHODS

### Ethical approval

All animal procedures complied with national and institutional regulations for the care and use of animals in research and followed ARRIVE 2.0 guidelines. This observational feeding trial was carried out on a farm and involved only routine husbandry and manual milking. No procedures beyond standard management were performed. Formal ethical approval was not required under the regulations of the Colegio de Postgraduados. The farm owner gave explicit consent for the study to be conducted on the premises and for handling the animals.

### Study period and location

The study was conducted from October to December 2022 at a private dairy goat farm located in the ejido Ignacio Zaragoza, Municipality of Viesca, Coahuila, within the Comarca Lagunera region of northern Mexico (24° N, 104° W; 1,100 m above sea level). The local climate is desert, semi-warm, with cool winters. The average annual air temperature (measured in shade) is about 25°C, and the average yearly precipitation is roughly 240 mm. The trial included a 14-day adaptation period followed by a 42-day experimental phase (Table 1).

**Table 1:** The sampling schedule across the 42-day experimental period.

Week (experimental)	Day range	Milk sampling	Forage sampling
1	1–7	Day 7 (06:00 a.m.)	Day 7
2	8–14	Day 14 (06:00 a.m.)	—
3	15–21	Day 21 (06:00 a.m.)	Day 21
4	22–28	Day 28 (06:00 a.m.)	—
5	29–35	Day 35 (06:00 a.m.)	—
6	36–42	Day 42 (06:00 a.m.)	Day 42

### Animals: Genotype, age, parity, health status, and body condition score (BCS) balance

All does were native “local” goats (Creole-type crossbreds adapted to arid rangelands) typical of the Comarca Lagunera production basin. Genotype strain identity was confirmed [Alpine × Saanen × Toggenburg] by phenotypic criteria used locally for Creole goats (ear length/shape, coat patterns, compact body size, and horn status) and herd origin, consistent with published descriptions of Creole goats in Mexico.

At enrollment (early-lactation), the participant met the inclusion criteria of being clinically healthy, non-pregnant, and within a narrow physiological window for the stage of lactation (days in milk [DIM]: [–] days). Animals were blocked by live weight, parity, and BCS, then randomly assigned to treatments (housed vs. grazing), ensuring comparable baseline status across groups, as previously indicated for weight and parity in your file. Age and parity. Age ranged from 2 to 3 years (mean ± SD: 2.3 ± 0.4). The full parity distribution per group is as follows:

**Table:** 

Group	n	Parity 2	Parity 3
Housed	5	4	1
Grazing	5	3	2

The BCS (1–5 scale, 0.25 increments) was evaluated by the same trained evaluator; goats were balanced so that the baseline BCS distributions were comparable between groups. Baseline BCS (mean ± SD): housed 3.3 ± 0.7 vs. grazing 3.4 ± 0.9; initial between-group difference: p = 0.627.

All animals received a physical examination, including checks of rectal temperature, respiratory and heart rates, rumen motility, and gait, along with udder inspection. Deworming and vaccinations were carried out according to the farm’s schedule. Exclusion criteria included fever, clinical mastitis, lameness, systemic disease, recent antibiotic therapy (<30 days), or BCS outside [–]. No replacements took place after the trial began.

### Production systems

#### Housed system

The animals allocated to the housed treatment were kept in individual pens measuring 2 × 3 m with shade, individual feeders, and waterers, with water available ad libitum. The base diet consisted of alfalfa hay (40%), oat hay (25%), and a commercial concentrate (35%) on a dry matter (DM) basis (Table 2). Bedding was straw and was replaced weekly. The ration was offered twice daily at 09:00 a.m. and 16:00 p.m., with daily refusals recorded to estimate individual intake. The concentrate was a milled dairy-goat feed (12% crude protein (CP); Lagunero®, Torreón, Mexico).

**Table 2:** Ingredients and chemical composition of animal diets.

Housed goats diet							
Ingredients							(Inclusion %)
Alfalfa hay							40
Oat hay							25
Concentrated feed							35

Chemical composition							(DM %)
Dry matter							94.56
Organic matter							91.47
Ashes							8.53
Crude protein							12.77
Ethereal extract							1.76
Neutral detergent fiber							28.48
Acid detergent fiber							27.15

**Grazing goats diet**							

**Forage**	**DM (%)**	**OM (%)**	**ASHES (%)**	**CP (%)**	**EE (%)**	**NDF (%)**	**ADF (%)**

*Chloris virgata*	94.39	87.72	12.28	4.73	1.16	33.57	32.49
*Cynodon dactylon*	94.34	90.51	9.49	6.59	1.10	32.29	30.56
*Parthenium bipinnatifidum*	93.59	85.37	14.63	18.41	6.02	14.04	13.16
*Acacia spp.*	93.96	89.76	10.24	18.98	5.44	18.60	17.95
*Chenopodium berlandieri*	94.95	83.99	16.01	14.91	2.82	28.97	26.75
*Solanum elaeagnifolium*	95.12	87.58	12.42	12.22	2.96	34.07	28.59

DM = Dry matter, OM = Organic matter, CP = Crude protein, EE = ethereal extract, NDF = Neutral detergent fiber, ADF = Acid detergent fiber.

#### Grazing system

The assigned grazing period was from 12:00 p.m. to 6:00 p.m. on native rangeland surrounding the farm, covering 4–8 km daily along a fixed route in the afternoon. The sward included *Chloris virgata*, *Cynodon dactylon*, *Parthenium bipinnatifidum*, *Acacia spp*., *Chenopodium berlandieri*, and *Solanum elaeagnifolium* (Table 2).

Forage availability during the trial averaged 35 kg DM ha^−1^ (estimated by double-sampling), with a stocking density of four goats ha^−1^ over the 60 ha grazed area. The grazing route and paddock sequence remained consistent across days to reduce plant-selection variability. Both groups had free access to minerals and vitamins via a mineral block placed in each pen and at the pasture water point.

#### Adaptation and experimental period

All goats underwent a 14-day adaptation to housing, grazing routines, and diets, followed by a 42-day experimental period for data collection. Milk sampling. Goats were manually milked at 06:00 a.m., and milk yield was recorded weekly for each animal during the 42 days. Individual 100-mL milk samples were collected once per week (mid-milking) and stored at 20°C for composition analysis. Forage sampling. During the grazing route, approximately 300 g of each forage species consumed was sampled for chemical analysis. Forage was sampled in weeks 1, 3, and 6, and composite samples were prepared for each species after grinding to 1 mm for laboratory analysis.

### Botanical composition, sampling method, phenology, and diet selection

The grazed rangeland consisted of *C. virgata*, *C. dactylon*, *P. bipinnatifidum*, *Acacia spp*., *C. berlandieri*, and *S. elaeagnifolium*. Botanical composition was estimated using quadrat sampling: 0.5-m² quadrats were systematically placed along the grazing route each week; species-level biomass was clipped at around 2 cm, oven-dried (60°C, 48 h), and expressed as g DM m^−2^.

Sampling took place during the early dry season when the stand was mostly mature: grasses were actively tillering, and forbs and shrubs were leafy and flowering. This phenological context is important for understanding the FA profile and protein content of the forage offered.

Bite-count and focal-scan observations (10-min bouts per goat; five goats per group; six observation days) revealed a higher preference for *Accacia* spp. and *C. virgata* inflorescences and avoidance of *C. dactylon*. Therefore, chemical analyses focused on the species that were preferred (composite by preference).

### Milk harvesting, hygiene, yield measurement, and composition analysis

All goats were hand-milked at 06:00 a.m. by trained personnel using clean, dedicated stainless-steel buckets; teats were dry-wiped to remove debris, pre-dipped with iodine (0.5%), foremilk discarded, and dried with single-use towels. Milking utensils were washed and sanitized. The individual morning yield (06:00 a.m.) was recorded weekly using a portable scale (Torrey® EQB, Mexico, 10 kg, ±1 g). The herd is milked once daily; therefore, the recorded morning yield equals the daily yield (no extrapolation).

A 100-mL composite sample was collected from each goat during mid-milking and stored at 20°C until analysis. Fat, protein, and lactose were quantified by infrared spectrophotometry (MilkoScope Expert Analyzer, Rasgard, Bulgaria) at the INIFAP dairy laboratory (Matamoros, Coahuila). Measurements followed the Association of Official Analytical Chemists Official Methods (AOAC, 2005) [9], with instrument calibration against certified standards at the start of each batch.

Each milk sample was analyzed in duplicate or triplicate, and the batch was re-run if the relative percentage difference exceeded 5%. Laboratory quality-control measures included blanks and reference milk.

FCM 3.5% was calculated as: FCM = 0.43 × milk (kg) + 16.23 × fat (kg)

### Feed analysis

The chemical compositions of concentrate and forage samples included DM, organic matter (OM), CP, ether extract (EE) [9], NDF, and ADF [10]. Additionally, the FA profile of the base diet and the consumed forage species was determined (Table 3). During grazing, 300 g of forage was collected blindly with respect to treatment and ground in a Thomas WILEY mill (Model 4, 1-mm mesh) along with the base diet for analysis. Three plant samples consumed by goats were collected during the first, third, and sixth grazing weeks, and a composite sample was prepared for each species to represent the overall chemical composition. FA extraction was performed according to the methodology of Feng *et al*. [11]. The FA profile was determined using the modified methylation technique of Palmquist and Jenkins [12] and Granados-Rivera *et al*. [13], in which FAs are in the form of methyl esters.

**Table 3:** FA profile (g/100 g FA) of the experimental animals’ base diet and forage species.

Basal diet	*Chloris virgata*	*Cynodon dactylon*	*Parthenium bipinnatifidum*	*Acacia spp.*	*Chenopodium berlandieri*	*Solanum elaeagnifolium*
0.82	2.68	2.14	0.95	2.14	0.53	0.75
0.71	2.97	1.56	0.95	2.05	0.42	1.09
ND	0.85	ND	0.61	0.16	0.18	0.24
24.60	38.18	27.17	25.14	25.74	24.36	21.75
0.19	1.44	ND	0.17	ND	0.22	0.43
0.17	1.02	0.56	0.21	0.59	0.32	0.80
3.37	4.85	3.88	2.91	8.88	3.61	6.18
0.31	1.17	ND	ND	ND	ND	ND
17.76	6.65	13.17	3.21	3.39	9.63	3.88
38.70	18.60	24.73	23.28	11.66	26.54	26.41
8.54	8.56	17.39	28.20	32.09	26.36	30.98
0.50	3.67	2.92	1.08	1.91	0.75	0.98
0.29	3.08	1.66	0.82	0.66	1.07	1.02
ND	ND	ND	0.10	1.15	0.70	0.41
0.36	2.59	1.88	1.88	0.72	1.26	0.93

ND=Not detected, FA = Fatty acid

For forage and concentrate samples, 0.5 g was placed in 50-mL culture tubes with Bakelite caps. Sodium methoxide (0.5 M in methanol) was added (3 mL) and gently vortexed. For milk samples, 25 mL was centrifuged at 12,000 × *g* for 30 min at 4°C. Lipids (50 μL) were transferred to tubes and centrifuged at 13,000 × *g* for 20 min; 0.2 g of the upper phase was then placed in 50-mL culture tubes. Sodium methoxide (2 mL) was added and vortexed. All tubes were incubated in a 50°C water bath for 10 min, cooled for 5 min, and then 3 mL of 5% methanolic hydrochloric acid was added and vortexed. The samples were heated in an 80°C water bath for 10 min and cooled for 7 min. Hexane (3 mL) was added to extract fat, followed by the addition of 6% potassium carbonate (5 mL) to saponify and neutralize. Samples were centrifuged at 3500 × *g* for 10 min. The organic phase was transferred to tubes containing 0.5 g of sodium sulfate and 0.5 g of activated carbon, vortexed, and centrifuged at 2500 × *g* for 10 min. The final extract was filtered through an Acrodisc (0.45-μm nylon membrane) and stored at –20°C.

FA methyl esters were analyzed using a Hewlett-Packard 6890 GC (Hewlett-Packard 6890, USA) chromatograph with an automatic injector and a silica capillary column (100 m × 0.25 mm × 0.20 μm, SP-2560, Supelco) with helium as the carrier gas. Peaks were identified by comparing retention times with a Supelco 37-component FA standard. Protein, fat, and lactose in milk were also measured using infrared spectrophotometry with the MilkoScope Expert Analyzer (Rasgard, Bulgaria) on 100-mL individual samples.

### Statistical analysis

Data were analyzed using a completely randomized design with repeated-measures. The experimental unit was the individual doe. Repeated measurements were taken weekly (6 time points; Days 7–42) for milk yield and composition; FA variables followed the same sampling weeks.For each response variable y (milk yield, fat, protein, lactose, and individual FA), the linear mixed model was:

yijk = μ + Ti + Wj + (T×W)ij + Ak(i) + εijk

Where Ti = treatment (housed, grazing), Wj = week (1–6), T×W = interaction, Ak(i) = random effect of doe nested within treatment.

The repeated structure was specified on a week within a doe, and the covariance structure (compound symmetry, autoregressive, unstructured, or Toeplitz) was selected using the lowest Akaike Information Criterion. Results are reported as least-squares means (Mean ± Standard error).

SAS v9.4 (SAS Institute Inc., Cary, NC, USA) was used, employing PROC MIXED. Residual normality was evaluated using the Shapiro–Wilk test on studentized residuals, and variance homogeneity was also checked. When necessary, transformations (log, square-root, or logit for proportions) were applied and refitted, with inferences made on the transformed scales and back-transformed means.

Pairwise differences among treatments within each week and overall means were tested using Tukey–Kramer adjustments at p < 0.05. Schwarz Bayesian Information Criterion (BIC) and AIC were used to identify the most suitable covariance structure. A variance-component procedure was applied for the repeated-measures design.

## RESULTS

### Milk production, FCM, and chemical composition

Milk production, FCM, and chemical composition showed significant differences between treatments (p < 0.05) (Table 4). Milk yield and FCM increased by 74.1% and 47%, respectively, in housed goats compared to grazing goats (p < 0.05).

**Table 4:** Milk production and composition from local housed and grazing goats.

Variables	Treatments		SEM	p-value		
	
	Housed	Grazing		Trat	Time	Trat*Time
Milk production (kg d^-1^)	1.41^a^	0.81^b^	0.09	0.0025	<0.0001	0.0048
Corrected milk production (3.5% fat kg d^-1^)	1.22^a^	0.83^b^	0.12	0.0549	0.0007	0.4832
Milk composition (%)						
Fat	2.72^b^	3.87^a^	0.29	0.0238	0.0056	0.6214
Protein	3.39^a^	2.74^b^	0.09	0.0011	0.0151	<0.0001
Lactose	5.02^a^	4.13^b^	0.16	0.0041	0.0128	0.0002

**Variables**	**Treatments**		**SEM**	**p-value**		
	
	**Housed**	**Grazing**		**Trat**	**Time**	Trat*Time

Milk yield (g d^-1^)						
Fat	37.85	29.90	5.12	0.3038	0.1061	0.9596
Protein	47.95^a^	21.85^b^	3.56	0.0008	<0.0001	0.1415
Lactose	71.11^a^	33.02^b^	5.31	0.0010	<0.0001	0.0926

^ab^Different letters between columns indicate a difference (p <0.05). SEM = Standard error of the mean.

**Figure 1:**
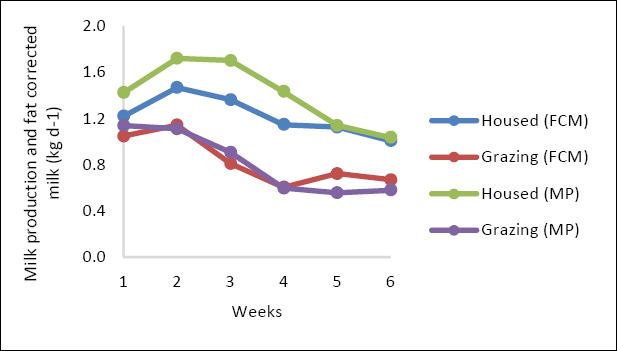
Milk production and fat-corrected milk in housed and grazing goats over time.

**Figure 2:**
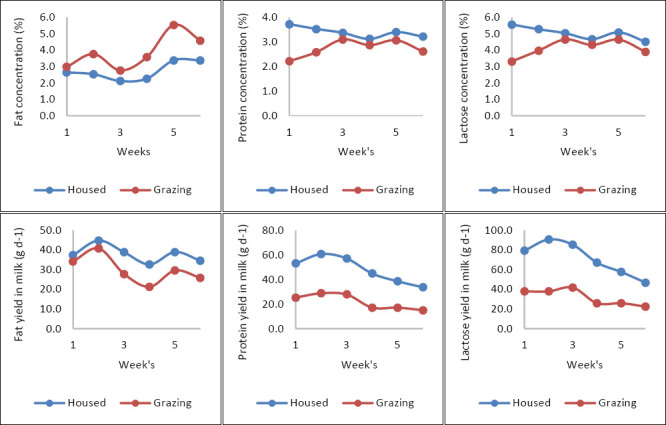
Chemical composition of local goat milk in housed and grazing systems over time.

As lactation progressed, daily milk production declined, and FCM at 3.5% showed a similar decreasing trend (Figure 1). The milk-fat concentration stayed relatively stable throughout the experimental period, with a slight rise toward the end. In both treatments, the protein and lactose concentrations and yields decreased over time (p < 0.05). Milk-fat yield did not significantly differ between treatments (p > 0.05) (Figure 2).

The milk-fat concentration was 42.3% higher in grazing goats (p = 0.0238). The protein concentration was higher in milk from housed goats, showing a 23.7% increase compared with grazing goats (p < 0.05). Lactose production was significantly higher in the milk of housed goats (p = 0.0041), representing a 21.54% increase relative to grazing goats.

### Milk FA profile

The FA profile of milk varied significantly between production systems (Table 5). The most common milk FAs in both systems were capric acid (C10:0), lauric acid (C12:0), myristic acid (C14:0), palmitic acid (C16:0), stearic acid (C18:0), and oleic acid (C18:1 c-9), together accounting for 85.2% of total FAs in housed goats and 77.3% in grazing goats.

**Table 5:** Fatty acid profile (g/100 g of total FA) of milk from local goats in housed and grazing systems.

Fatty acid	Treatments		SEM	p-value		
	
	Housed	Grazing		Trat	Time	Trat*Time
C6:0	3.20^a^	2.67^b^	0.14	0.0237	0.0004	0.0003
C8:0	3.70^a^	2.76^b^	0.11	0.0003	0.0157	<0.0001
C10:0	14.50^a^	9.89^b^	0.26	<0.0001	0.0305	<0.0001
C11:0	0.41^a^	0.28^b^	0.02	0.0033	0.0046	0.0004
C12:0	6.81^a^	4.07^b^	0.18	<0.0001	0.0021	<0.0001
C13:0	0.21^a^	0.12^b^	0.01	0.0007	0.0259	0.0222
C14:0	12.95^a^	10.16^b^	0.36	0.0005	<0.0001	<0.0001
C14:1	0.10	0.09	0.01	0.4339	0.0115	0.1429
C15:0	1.08	1.15	0.06	0.4256	<0.0001	<0.0001
C16:0	30.73^a^	25.44^b^	1.18	0.0133	<0.0001	<0.0001
C16:1	0.87	0.88	0.04	0.9038	<0.0001	0.0026
C17:0	0.77^b^	0.95^a^	0.03	0.0032	<0.0001	0.0007
C17:1	0.20^b^	0.31^a^	0.02	0.0029	0.0001	0.0100
C18:0	6.56^b^	8.70^a^	0.50	0.0163	0.0085	0.0316
C18:1 *t*9	0.74^b^	1.34^a^	0.08	0.0006	<0.0001	0.0002
C18:1 *c*9	13.67^b^	19.03^a^	0.92	0.0033	0.0023	0.0742
C18:2 *c*9, *c*12	1.68^a^	1.29^b^	0.08	0.0089	0.0012	0.2915
C18:2 *c9, t*11 CLA^1^	0.20^b^	0.46^a^	0.36	0.0009	0.0008	0.3667
C18:3 n-3	0.29^b^	0.43^a^	0.03	0.0143	0.0085	0.6594
C20:0	0.13^b^	0.26^a^	0.01	<0.0001	<0.0001	0.0002
C20:4	0.07	0.06	0.01	0.3230	0.0003	0.1721
Total UN	0.84^b^	1.09^a^	0.05	0.0085	0.0043	0.0064
Total SFA^2^	81.25^a^	66.44^b^	1.63	0.0002	<0.0001	<0.0001
Total MUFA^3^	15.86^b^	21.70^a^	0.91	0.0018	0.0005	0.0627
Total PUFA^4^	2.22	2.25	0.13	0.8757	0.0011	0.4381
Σ <C16:0	42.99^a^	31.18^b^	0.74	<0.0001	0.0033	<0.0001
Σ C16:0 + C16:1	31.61^a^	26.38^b^	1.19	0.0147	<0.0001	<0.0001
Σ >C16	24.59^b^	32.84^a^	1.42	0.0034	0.0010	0.0547

^a b^ Different letters between columns indicate a difference (p < 0.05). SEM = Standard error of the mean, UN = Unidentified,

1 CLA = Conjugated linoleic acid,

2 SFA = Sum of saturated FAs,

3 MUFA = Sum of monounsaturated FAs,

4 PUFA = Sum of polyunsaturated FAs, FAs = Fatty acids.

Compared to milk from housed goats, milk from grazing goats showed higher levels of C18:1 c-9, C18:1 t-9, cis-9, trans-11 CLA, C18:3 n-3, and several long-chain saturated FAs (SFAs). Conversely, grazing lowered the levels of medium-chain SFAs, including C10:0, C12:0, and C14:0.

### Treatment × time interactions for FAs

**Figure 3:**
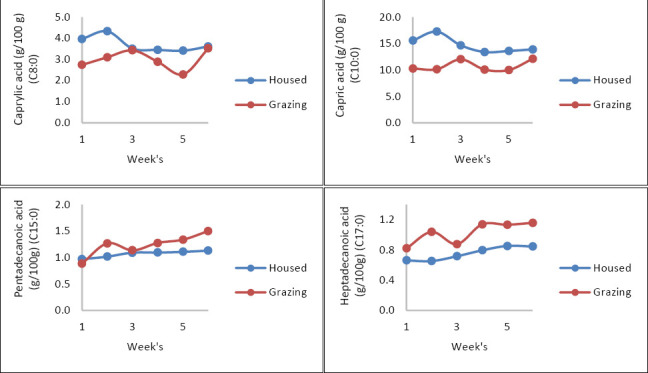
Fatty acids of milk from local goats of dietary importance in a housed and grazing system over time.

Significant treatment × time interactions were observed for several FAs, notably C8:0, C10:0, C15:0, and C17:0 (Figure 3). For C8:0 and C10:0, increases became more noticeable starting from the third week of the experiment, with higher values seen in the milk of housed goats.

In contrast, C15:0 and C17:0 showed rising trends from the beginning to the end of the experiment, with higher levels in the milk of grazing goats. These patterns emphasize the impact of the production system and lactation stage on milk FA composition.

## DISCUSSION

### Milk production and FCM: Metabolic and dietary drivers

The increase in milk and FCM production in housed goats compared to grazing goats is due to concentrate intake, which raises ruminal propionate levels. Propionate is converted into glucose in the liver and then into lactose in the mammary gland [14], causing more water to move into the mammary secretory cells and resulting in higher milk volume [15]. Milk production decreases during grazing because of the extra energy used for displacement during foraging and muscle activity involved in grasping, chewing, and moving the feed [16]. Shinde and Karim [17] reported that the maintenance energy requirement in goats is 424–522 kJ kg^−1^ higher than in confined animals. Additionally, the lower milk production during grazing is directly related to the limited availability and quality of forage, which does not meet nutritional needs and leads to an energy and protein shortfall [18]. This forces goats to mobilize their body reserves [19], resulting in protein being drawn from muscle fibers for milk production and, consequently, a reduction in milk yield [20].

### Milk-fat concentration and rumen fermentation dynamics

Milk-fat concentration was 42.3% higher in grazing goats (p = 0.0238). This increase is attributed to higher fiber intake, which is essential for proper rumen function and saliva production. Saliva enhances ruminal pH through its buffering capacity, promoting the growth of cellulolytic microorganisms such as *Fibrobacter succinogenes, Ruminococcus flavefaciens*, and *Ruminococcus albus* [21]. These microorganisms produce high levels of acetic, butyric, and isobutyric acids, which are primary precursors of milk-fat [22]. Bromatological analysis of the forage consumed by the animals showed that grazing goats consumed 45.85% more lipids than housed goats. According to D’Urso *et al*. [23], forages contain higher concentrations of galactolipids, especially linoleic acid (C18:2 c9, c12) and α-linolenic acid (C18:3 n-3), which are hydrolyzed into glycerol, FAs, and galactose. The galactose is rapidly fermented and converted into volatile FAs. Consequently, the higher milk-fat production in grazing goats was also linked to increased production of acetic acid, a key precursor of milk-fat [18, 22, 23].

### Milk protein concentration: Effects of energy availability and mammary signaling

Milk protein concentration was higher in housed goats (p = 0.0011), showing a 23.7% increase compared to grazing goats (p < 0.05). This difference is mainly due to concentrate intake, where carbohydrates are easily fermentable, producing greater energy availability, propionic acid, and microbial protein [24]. These processes cause metabolic changes in the animal, including increased circulating insulin, which affects mammary gland physiology and enhances protein synthesis, leading to higher protein levels in the milk of housed goats [25].

### Lactose production and glucose availability

Lactose production was higher in housed goats (p = 0.0041), showing a 21.54% increase compared to pastured goats. This contradicts Lin *et al*. [26], who suggested that lactose is the most stable component in ruminant milk and is generally unaffected by dietary changes [27]. However, concentrated feeds produce higher levels of ruminal propionic acid [28], which serves as the main substrate for glucose production [29], the primary precursor for lactose synthesis in mammary epithelial cells [15]. This metabolic pathway explains the increased lactose production in milk from stall-fed goats. A similar pattern was reported by Monzón-Gil *et al*. [27], who linked higher lactose production to increased overall milk yield.

### Lactation progression and changes in milk components

The results of milk production followed a typical lactation curve [30], where daily milk production decreased as lactation progressed due to physiological interactions involving apoptosis of milk-secreting cells, growth hormone, and prolactin [15], all of which decline with advancing lactation. FCM at 3.5% followed a similar trend, remaining near the significance threshold (p = 0.0549). Milk-fat concentration stayed stable with a slight increase toward the end of the experimental period. Protein and lactose concentrations and their yields decreased over time in both treatments (p < 0.05), while milk-fat yield was not significantly affected (p > 0.05). Isidro-Requejo *et al*. [31] observed a similar pattern in local goats in the Comarca Lagunera. Martin *et al*. [32] reported that as milk production decreases, fat content may increase due to concentration effects, whereas fat concentration drops during periods of higher milk yield due to a dilution effect.

### FA profile differences between systems

The differences in milk FA(FA) profiles between production systems confirm earlier findings in local goats during late-lactation under arid conditions [6, 7, 23]. The predominance of capric, lauric, myristic, palmitic, stearic, and oleic acids remains characteristic of goat milk [33]. Elevated cis-9, trans-11 CLA and n-3 FA concentrations in grazing goats highlight the nutritional benefits of extensive systems. Conversely, confinement maximizes milk volume and protein-rich solids, illustrating a trade-off that producers need to consider based on market objectives and consumer health demands.

Milk’s natural bioactive components and its enrichment potential can be used to create high-quality, health-oriented products aimed at nutraceutical markets. These products attract health-conscious consumers looking for better digestibility or improved FA profiles and may fetch higher prices.

### Implications for production systems, sustainability, and consumer markets

Grazing systems are often preferred in markets that prioritize animal welfare, sustainability, and high-quality milk over maximum production. Partial supplementation strategies can compensate for lower milk yields by enhancing nutritional balance and boosting efficiency through targeted nutrient supply. Additionally, this study supports climate change adaptation strategies for smallholder systems in semi-arid Mexico by showing how environmental variability influences animal performance and by identifying existing adaptation practices and challenges. Combining local and scientific knowledge, such as using drought-tolerant forages and adjusting planting cycles, with financial and institutional support is crucial for strengthening system resilience.

### Opportunities for integrated FA pathway analysis

A comprehensive FA profiling approach that combines forage, concentrate, and milk analysis would illustrate the complete nutritional pathway. Such an approach would trace how polyunsaturated FAs, including C18:2 and C18:3, present in forage, and monounsaturated FAs like C18:1 in concentrates, are modified through ruminal biohydrogenation and microbial synthesis before being incorporated into milk-fat. This integrated framework would show how the forage-to-concentrate ratio influences the final milk FA profile, high-forage diets typically increase beneficial FAs such as CLA and omega-3s, while high-concentrate diets may decrease milk-fat content but promote body weight gain. These insights offer a holistic understanding of how dietary components impact milk nutritional quality.

## CONCLUSION

This study clearly shows that the production system significantly influences milk yield, composition, and FA profiles in native goats managed under semi-arid conditions. Housed goats produced much higher milk and FCM yields, 74.1% and 47% greater, respectively, mainly due to increased propionate production from concentrate-rich diets and its role in glucose and lactose synthesis. In contrast, grazing goats had significantly higher milk-fat concentration (+42.3%), mainly supported by higher fiber intake, better ruminal buffering, and increased fermentation to acetic and butyric acids by cellulolytic microorganisms such as *F. succinogenes*, *R. flavefaciens*, and *R. albus*. Grazing also resulted in higher levels of nutritionally valuable FAs, including cis-9, trans-11 CLA and omega-3 polyunsaturated FAs, emphasizing the better nutritional profile linked to extensive systems.

These findings have significant practical implications for producers and processors. Indoor systems are beneficial when the main goal is to maximize milk volume and protein-rich solids, which are vital for fluid milk markets and cheese manufacturing. Grazing systems, on the other hand, produce milk with more desirable health-promoting FAs, creating opportunities for specialty, premium, or functional dairy products that attract health-conscious consumers. Adjusting production strategies to match market demands, either by leveraging the natural advantages of grazing or by adding partial supplementation to boost both yield and quality, can improve profitability, sustainability, and product differentiation, especially for smallholder farms in dry regions.

The strength of this study lies in its controlled comparison of two contrasting production systems using native goats specifically adapted to desert environments. By assessing milk yield, proximate composition, and a detailed FA spectrum during early-lactation, the study provides an integrated dataset that is rarely available for local Mexican goat populations. However, limitations such as the small sample size, single-season evaluation, and lack of extended lactation monitoring diminish the generalizability of the results and may not fully reflect temporal or seasonal variability.

Future research should include larger herds, multi-seasonal sampling, and mid- to late-lactation stages to better understand temporal patterns. Mechanistic studies linking forage composition, ruminal biohydrogenation pathways, and milk FA deposition would further improve understanding of how diet influences milk nutritional quality. Evaluating partial supplementation strategies, drought-resilient feeding systems, and economic feasibility models will also be crucial to support climate-adaptive and resource-efficient production under semi-arid conditions.

In conclusion, this study offers valuable evidence that both grazing and confinement have unique strengths: grazing improves milk nutritional quality, while confinement maximizes yield and protein content. By strategically combining these benefits, producers can enhance resilience, promote animal welfare, and produce milk that meets diverse market and consumer demands in arid and semi-arid regions.

## DATA AVAILABILITY

The supplementary data can be made available from the corresponding author upon request.

## AUTHORS’ CONTRIBUTIONS

MTTL and OHM: Designed and conducted the study and drafted and edited the manuscript. GTH and DHS: Coordinated and guided the research. LDGR and JAMJ: Data collection, statistical analysis, and laboratory methods. All authors have read and approved the final version of the manuscript.
